# UPPER MIDLINE INCISION IN RECIPIENTS OF DECEASED-DONORS LIVER TRANSPLANTATION

**DOI:** 10.1590/0102-672020180001e1389

**Published:** 2018-08-16

**Authors:** Olival Cirilo Lucena da FONSECA-NETO, Américo Gusmão AMORIM, Priscylla RABELO, Heloise Caroline de Souza LIMA, Paulo Sérgio Vieira de MELO, Cláudio Moura LACERDA

**Affiliations:** 1 University Hospital Oswaldo Cruz, Faculty of Medical Sciences of Pernambuco, University of Pernambuco, Recife, PE, Brazil.

**Keywords:** Upper midline incision, Liver transplant, Transplant recipients, Incisão mediana superior, Transplante de fígado, Transplantados

## Abstract

**Background:**

Liver transplant (LT) is the only effective and long-lasting option for patients with end-stage liver disease. Innovations and refinements in surgical techniques occurred with the advent of transplants with partial grafts and laparoscopy. Despite these modifications, the abdominal incision remains with only few changes.

**Aim:**

Demonstrate the experience with the upper midline incision in LT recipients with whole liver grafts from deceased donors.

**Methods:**

Retrospective study with patients submitted to LT. Data were collected from the recipients who performed the surgical procedure through the upper midline incision.

**Results:**

The upper midline incision was used in 20 LT, 19 of which were performed in adult recipients. The main cause was liver disease secondary to alcohol. Male, BMI>25 kg/m² and MELD greater than 20 were prevalent in the study. Biliary complications occurred in two patients. Hemoperitoneum was an indication for reoperation at one of the receptors. Complication of the surgical wound occurred in two patients, who presented superficial surgical site infection and evisceration (omental). Two re-transplant occurred in the first postoperative week due to severe graft dysfunction and hepatic artery thrombosis, which were performed with the same incision, without the need to increase surgical access. There were two deaths due to severe graft dysfunction after re-transplant in 72 h and respiratory sepsis with multiple organ dysfunction in the third week.

**Conclusion:**

The upper midline incision can be safely used in LT recipients with whole grafts from deceased donors. However, receptor characteristics and hepatic graft size should be considered in the option of abdominal surgical access.

## RESUMO

**Racional:**

O transplante de fígado (TF) é a única opção eficaz e duradoura para os pacientes com doença hepática em estágio terminal. Inovações e refinamentos nas técnicas cirúrgicas ocorreram com o advento dos transplantes com enxertos parciais e da laparoscopia. Apesar dessas modificações, a incisão abdominal permanece sem grandes mudanças.

**Objetivo:**

Demonstrar a experiência com a incisão mediana superior nos receptores de TF com enxertos hepáticos inteiros provenientes de doadores falecidos.

**Método:**

É estudo retrospectivo entre os pacientes submetidos ao TF. Foram coletados os dados dos receptores que realizaram o procedimento cirúrgico através da incisão mediana superior .

**Resultados:**

Essa incisão foi utilizada em 20 TF, sendo 19 realizados em receptores adultos. A principal causa foi a doença hepática secundária ao álcool. O gênero masculino, IMC>25 kg/m² e o MELD superior a 20 foram prevalentes no estudo. Complicações biliares ocorreram em dois pacientes. Hemoperitônio foi indicação de reoperação em um dos receptores. Complicação da ferida cirúrgica ocorreu em dois pacientes, que apresentaram infecção de sítio cirúrgico superficial e evisceração (omental). Ocorreram dois re-transplantes na primeira semana de pós-operatório devido à disfunção grave do enxerto e à trombose da artéria hepática, sendo realizados com a mesma incisão, sem a necessidade de ampliar o acesso cirúrgico. Ocorreram dois óbitos por disfunção grave do enxerto após o re-transplante em 72 h e sepse respiratória com disfunção de múltiplos órgãos na terceira semana.

**Conclusão:**

A incisão mediana superior pode ser utilizada com segurança em receptores de TF com enxertos inteiros provenientes de doadores falecidos. Entretanto, características do receptor e tamanho do enxerto hepático devem ser considerados na opção do acesso cirúrgico abdominal.

## INTRODUCTION

Liver transplantation (TF) is the only effective and long-term option for patients with end-stage liver disease. The surgical procedure is complicated and challenging, making it one of the most complex operations performed today in humans. After 50 years, TF evolved with an improvement in the survival of liver receptors and grafts thanks to the greater knowledge in multiple areas such as anesthesia, intensive therapy, immunology and surgical technique.

Innovations and refinements in surgical techniques occurred with the advent of transplants with partial grafts (reduced graft, between live donors and split-liver grafts), as well as laparoscopy, especially in liver resections, extending to hepatectomies in living donors (right or left). Despite these modifications, the abdominal incision for the TF remains unchanged.

The aim of this study was to demonstrate the results with the upper midline (supra-umbilical) incision in TF recipients with whole liver grafts from deceased donors.

## METHODS

It is a retrospective study among patients submitted to TF from August 1999 to June 2016 at the General Surgery and Hepatic Transplantation service at Oswaldo Cruz University Hospital, Recife, PE, Brazil. Only the recipients who performed the surgical procedure through the median upper incision were evaluated. Demographic data were collected through the service database, through which follow-up and all complications were identified. The Clavien-Dindo classification of surgical complications was used. Post-operative mortality was defined as death within 90 days of the operation.

The conventional TF technique without venous deviation was obeyed without any changes in all procedures. The medial incision extended from the lower end of the xiphoid appendix to the upper portion of the umbilical scar. The placement of the retractors was not modified and no transformation in this instrument occurred for re-incision purposes. Antibiotic prophylaxis was performed with piperacillin-tazobactan and abdominal drainage was routinely performed with silicone device No. 24.

Immunosuppression was standardized for all receptors consisting of a triple scheme: tacrolimus, prednisone, and mycophenolate mofetil. The possible withdrawal/replacement of these medications followed the outpatient course.

## RESULTS

The median upper incision was used in 20 TF of the 1067 performed between August 1995 and June 2016. All procedures were done on adult recipients, except for one, related to an eight-year-old girl diagnosed with Budd-Chiari syndrome, receiving a hepatic graft from a deceased pediatric donor of nine years, and progressing uneventfully.

The most present cause in this study was liver disease secondary to alcohol. The male gender and the BMI>25 kg/m² were also prevalent among those submitted to TF. Regarding the severity of liver disease, MELD (Model End-Liver Disease) was higher than 20 in most cases. Large ascites volume was frequent in the study patients (in three cases the drainage was 15 l). This drainage occurred during the opening of the abdominal cavity at the beginning of the surgical procedure.

As to liver weight in the recipient, 12 presented less than 1000 g, while the graft had>1000 g.

The surgical time was above 5 h. On three occasions, during the operation of the back-table, due to the need for multiple arterial reconstructions, the surgical team of the recipient waited 90 min.

The vascular access was performed by the anesthesiologist with the aid of ultrasound. This device remains in the operating room for use at any time during anesthesia/surgery. Despite this, pneumothorax on the right was found in one case related to vascular puncture, and thoracostomy was necessary under water seal removed in two days.

Blood loss was assessed by the amount of blood in the aspirator and the need for transfusion of blood components. Hemotransfusion was not required in more than half of the patients.

Extubation at the end of the TF occurred on eight occasions. Mechanical ventilation for more than 48 h was found in four: two who evolved to re-transplant and two with renal insufficiency dialysis and ventilatory sepsis.

Some complications were observed and classified according to the classification of Clavien-Dindo^3^ (Table 1).

**TABLE 1:** Complications observed in liver transplant recipients with superior median incision (2 to 24 months) using the Clavien-Dindo^3^ system

GRADE	COMPLICATION	n
I	Surgical wound infection Pleural effusion	n=1 n=3
II	Transfusion of blood components Postoperative ileus	n=8 n=1
IIIa	Bilioma Pneumothorax on the right	n=1 n=1
IIIb	Hemoperitoneum Biliary stenosis Evisceration	n=1 n=1 n=1
IVa	Hepatic graft dysfunction Renal dysfunction	n=1 n=3
IVb	Sepsis of respiratory origin	n=1
V	Death	n=2

Biliary complications occurred in two patients. Percutaneous drainage with drain placement was necessary and sufficient in one of them. Bilodigestive reconstruction (hepaticojejunoanastomosis) was performed in the patient who presented obstructive jaundice, in the 25^th^ day of development, evolving without intercurrences.

Dialytic renal insufficiency appeared in three TF receptors. It occurred within the first 48 h and was associated with increased blood loss and surgical time (>450 ml and >5 h, respectively).

Hemoperitoneum due to bleeding in the inferior vena cava (retrocaval region) was an indication for reoperation in one of the receptors in the 2^nd^ postoperative. It was not necessary to change the incision using the initial (median superior).

Complication of the surgical wound occurred in two patients, who presented superficial surgical site infection and evisceration (omental). Sanitization of the wound was sufficient in the first case. Regarding evisceration, it was necessary to re-operate to perform a new synthesis of aponeurosis.

Two re-transplants occurred in the first postoperative week of the primary procedure. These were also performed with the same median incision without the need to increase surgical access. Severe graft dysfunction and hepatic artery thrombosis were responsible for the need for new TF.

There were two deaths. The first, due to severe graft dysfunction necessitating re-transplantation and new organ dysfunction evolving to death within 72 h. The second, due to respiratory sepsis and multiple organ dysfunction and death in the 25^th^ day. All others are undergoing outpatient follow-up (Table 2).

**TABLE 2:** Data on liver transplantation recipients with upper median incision

n=18*	Receiver liver weight	Blood loss
Gender M/F: 11/7	> 1000 g-8	>450 ml-12
IMC (kg/m²)	<1000 g-12	<450 ml-8
>25-12		
<25-6		Extubation
MELD		SO-8
>20-12	Donor liver weight	<48 h-8
<20-6	<1000 g-8	>48 h-4
Ascites	> 1000 g-12	
>10l-14		UTI Time
<10l-4		>48 h-8
Etiology**	Surgical time	<48 h-12
VHC- 4	<5 h-14	
Alcohol-7	> 5 h-6	Hospital time
Criptogenic-3		<10 days -15
Autoimmune-1		>10 days-5
Budd-Chiar -2		
HCC-3		

BMI=body mass index; HCV=hepatitis C virus; HCC=hepatocellular carcinoma; SO=operating room; *eighteen patients underwent 20 procedures: two evolved to retransplantation;** some patients had more than one diagnosis

## DISCUSSION

**FIGURE 1:**
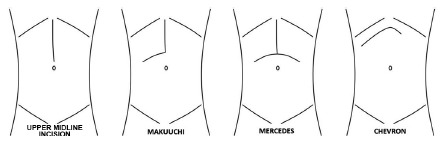
Major abdominal incisions used in liver transplantation

TF is classically performed with the following incisions: bilateral subcostal (Chevron), bilateral subcostal with xiphoid extension (Mercedes), or the J-shaped incision (Makuuchi)^1^ ^,^ ^6^ ^,^ ^10^ ^,^ ^11^ ^,^ ^18^ (Figure 1). With the advent of minimally invasive surgery, the option of smaller incisions contributed to better results. Although several authors reported their experiences with good results in neoplastic hepatectomies and in living donors^21^ ^,^ ^25^, the use of the medial supraumbilical incision in the TF recipient with whole graft from a deceased donor did not occur^15^.

Chang et al^2^ in their study on the use of modified Makuuchi incision (J-incision) in upper abdominal surgery described the excision of the xiphoid appendix as a way to optimize access, being performed routinely. Some authors reported incisional heterotypic bone formation after this practice^7^. In this study excision of the xiphoid appendix was not performed.

During hepatectomy of the native liver there is a need for dissection of the posterior segments near the diaphragm, adrenal gland and inferior vena cava. Thus, it is believed that the need for enlargement of the incision is to provide better surgical field in these areas. The safety and effectiveness of the midline incision in hepatic resections has been demonstrated for almost a decade^4^ ^,^ ^21^ ^,^ ^22^ ^,^ ^24^. A bleeding reoperation occurred in retrocaval space on the 2^nd^ postoperative day, without the need to modify the incision, and there were no significant repercussions in the graft or in the patient.

In patients with portal hypertension, the presence of collateral circulation in the abdominal wall may contribute to difficulties in the surgical access^8^. In horizontal incisions, a longer surgical time is required to perform hemostasis with electrocautery, and sometimes with ligature of the collateral vessels. This when occurs in the median incision is attenuated. Another characteristic found in these patients is the formation of hematoma in an operative wound. While transverse incisions appear frequently, medial incision is a less common concern^23^. There was no surgical wound hematoma in the studied patients.

Operative wound hypoesthesia, as well as postoperative pain, is a frequent complaint in TF receptors. This is due to the type of incision that does not save the innervation (incision Mercedes, Chevron, Makuuchi). The option for the supraumbilical median abdominal incision respects the innervation, with this leading to the lower postoperative pain and almost absence of hypoesthesia in the operative wound^14^ ^,^ ^21^ ^,^ ^24^ ^,^ ^28^. Respiratory complications are associated with upper (mainly subcostal) abdominal incisions and in TF is frequent. Three patients with postoperative pleural effusion were found (<20%).

The median supraumbilical incision is universally known, easy to perform and fast. It is a good option, mainly due to the postoperative evolution, the learning curve not long, less pain and less pleuropulmonary complications, without, however, compromising its safety, reproducibility or effectiveness^12^ ^-^ ^14^ ^,^ ^17^ ^,^ ^21^ ^,^ ^24^ ^,^ ^29^.

Although some authors use a technological tool to predict difficulty in surgery of the upper abdomen (depth ratio of the celiac artery), this did not occured in this study^17^. Similarly, some studies demonstrate the use of the median incision in live donor transplantation with the aid of optical fiber^27^. In the present study, laparoscopy was not required. Videolaparoscopy and laparoscopic procedures have been increasingly used in liver surgeries. Such procedures are expensive and require considerable experience of the practitioner in this art. Consequently, multiple attempts to reduce the surgical incision without the use of laparoscopy have been performed in the operations of living donors^16^ ^,^ ^19^ ^,^ ^20^ ^,^ ^27^. Shen et al^26^ in their study with 48 patients divided into two groups, using the upper midline incision with or without videolaparoscopic aid in liver transplantation involving right hemifer (donor operation), demonstrated that its use did not bring significant differences to the patient, besides increasing the hospital cost; they suggested the median incision as the first treatment line. This result was also demonstrated by other authors^17^ ^,^ ^29^.

Patient characteristics - BMI>25 kg/m^2^, large ascites volume, and explanted and implanted small liver - were the most prevalent findings that made the surgical procedure with the median incision easier. Intraoperative bleeding - despite being low volume in this study - and surgical time, may not be associated with the type of abdominal access, but with the surgical technique (conventional), as well as with the moment of extubation and hospital staying. However, it was observed in practice, that greater cooperation of the patients in the physiotherapeutic maneuvers (respiratory and motor) and less use of analgesics, contributed with shorter ICU time.

The type of incision depends on the surgeon’s choice and experience. The extent between the xiphoid appendix and the umbilical scar, as well as the body mass index of the recipient, are factors that contribute to the selection of the incision in these patients^4^. In obese and/or short stature receptors, is often difficult to find difficult dissections in the posterior segments^9^. However, some authors have demonstrated the possibility of this surgical time in the hepatectomy to be performed with the median incision, with caution and safety^21^.

The median incision was sufficient for the surgical access in the TF in all cases, and no modifications were necessary. Due to its anatomical topography, it saves the innervation and vascularization of the abdominal wall, contributing with a lower risk of wound complications, such as infection and dehiscence of aponeurosis^2^.

## CONCLUSION

The upper midline incision can be safely used in TF recipients with whole grafts from deceased donors. However, receptor characteristics and hepatic graft size should be considered in the option of abdominal surgical access.

## References

[1] Adani GL, Rossetto A, Bitetto D, Bresadola V, Baccarani U (2009). Which type of incisios for liver transplantation .Letter to the
editors.Liver. Transpl.

[2] Chang SB, Palavecino M, Wray CJ, Kishi Y (2010). Modified Makuuchi incision for foregut procedures. Arch Surg.

[3] Clavien PA, Barkun J, de Oliveira ML, Vauthey JN, Dindo D, Schulick RD (2009). The Clavien-Dindo classification of surgical complications
five-year experience. Ann Surg.

[4] Demirbas T, Buluctu F, Dayangac M, Yaprak O (2013). Which incision is better for living-donor right hepatectomy
Midline, J-Shaped or Mercedes. Transplant Proc.

[5] Dindo D, Dematines N, Clavien PA (2004). Classification of surgical complications a new purposal with
evaluation in a cohort of 6336 patients and results of a
survey. Ann Surg.

[6] Donataccio M, Genco B, Donataccio D (2006). Right subcostal incision in liver transplantation prospective
study of feasibility. Transpl Proc.

[7] Farnell MB (2010). Foregut surgery by the letter Is J better than Inverted T or V?.
Invited Critique. Arch Surg MAR.

[8] Fonseca-Neto OCL, Albuquerque-Neto MC, Miranda AL (2015). Tratamento cirúrgico da dilatação cística das vias biliares em
adulto. ABCD Arq Bras Cir Dig.

[9] Fonseca-Neto OCL, Lima HCS, Melo PSV, Lemos R, Leitão L, Amorim AG, Lacerda CM (2016). Apendicite aguda em receptores de transplante de
fígado. ABCD Arq Bras Cir Dig.

[10] Haberal M, Emiroglu R, Karakayali H, Moray G, Aslan G (2003). Exposure for hepatobiliary operations a new
incision. Transpl Proc.

[11] Heisterkamp J, Marsman HA, Eken H, Metselaar HJ, Tilanus HW, Kazemier G (2008). A J-Shaped subcostal incision reduces the incidence of abdominal
wall complications in liver transplantation Liver. Transpl.

[12] Ikegami T, Harimoto N, Shimokawa M, Yoshizumi T (2016). The learning curves in living donor hemi-liver graft procurement
using small upper midline incision. Clin Transplant.

[13] Israelsson LA, Millbourn D (2013). Prevention on incisional hérnias how to close a midline
incision. Surg Clin N Am.

[14] Jain A, Nemitz P, Sharma R, Sheikh B (2009). Incidence of abdominal wall numbness post-liver transplantation
and its complication. Liver Transpl.

[15] Kayaalp C, Aydin C, Unal B, Baskiran A (2011). Liver transplantation from an upper midline
incision. Exp Clin Transplant.

[16] Kurosaki I, Yamamoto S, Katami C, Yokoyama N, Nakatsuka H (2006). Video-assisted living donor hemihepatectomy through a 12cm
incision for adult-to-adult liver transplantation. Surgery.

[17] Kwang-woong L, Seong HK, Sung-Sin H, Young-Kyu K (2011). Use of na upper midline incision for living donor partial
hepatectomy a series of 143 consecutive cases. Liver Transpl.

[18] Lam H, Vanlander A, Berrevoet F (2016). A comparative outcome analysis of incisional hernia repair in
patients who underwent liver transplantation vs those that underwent
hepatopancreaticobiliary surgery using the EHS guidelines as a means of
comparison. Clin Transplant.

[19] Li H, Wei Y, Li B (2016). Total laparoscopic living donor right hepatectomy first case in
china mainland and literature review. Surg Endosc.

[20] Makki K, Chorasiya VK, Sood G, Srivastava PK, Dargan P, Vij V (2014). Laparoscopy-assisted hepatectomy versus conventional (open)
hepatectomy for living donos when you know better, you do
better. Liver Transpl.

[21] Manoj JS, Nir L, Matias F, Alan C (2016). Upper midline incision for living donor right
hepatectomy. Clin Transplant.

[22] Panaro F, Boisset G, Chanques G, Guiu B (2016). Vena cava encirclement predicts difficult native
hepatectomy. Liver Transpl.

[23] Rozen MW, Ashton MW, Taylor GI (2008). Reviewing the vascular supply of the anterior abdominal wall
redefining anatomy for increasingly refined surgery. Clin Anat.

[24] Seong HK, Seong YC, Kwang WL, Sang-Jae P, Sung-Sik H (2009). Upper midline incision for living donor right
hepatectomy. Liver Transpl.

[25] Seong HK, Young KK (2013). Upper midline incision for liver resection. HPB.

[26] Shen S, Zhang W, Jiang L, Yan L, Yang J (2016). Comparison of upper midline incision with and without
laparoscopic assistance for living-donor right hepatectomy. Transpl Proc.

[27] Shinoda M, Tanabe M, Itano O, Obara H, Kitago M, Abe Y (2014). Left-side hepatectomy in living donos through a reduced
upper-mildline incision for liver transplantation. Transpl Proc.

[28] Somaya A, Takatsuki M, Hidaka M, Adachi T (2015). Hybrid procedure in living donor liver
transplantation. Transplant Proc.

[29] Suh S, Lee KW, Lee JM, Choi Y (2015). Clinical outcomes of and patient satisfaction with diferente
incision methods for donor hepatectomy in living donor liver
transplantation. Liver Transpl.

